# Marital Status Disruptions and Internalizing Disorders of Children

**DOI:** 10.1155/2019/4634967

**Published:** 2019-06-09

**Authors:** Jordyn T. Wallenborn, Gregory Chambers, Elizabeth Lowery, Saba W. Masho

**Affiliations:** Virginia Commonwealth University, School of Medicine, Division of Epidemiology, Department of Family Medicine and Population Health, 830 East Main Street, Suite 821, P.O. Box 980212, Richmond, VA 23298-0212, USA

## Abstract

Marital disruption (i.e., separation or divorce) impacts an estimated 40-50% of married couples. Previous research has shown that marital disruption results in negative health outcomes for children and adolescents. Our study aims to investigate the relationship between marital disruptions and internalizing disorders of children in a prospective cohort. Comparisons between marital status groups at each time point showed a significant difference in CBCL score between children in married and unmarried families at 3 years of age, with children in unmarried families having a 0.10 higher standardized CBCL score (95% CI: 0.09-0.12; p<.0001). Differences in CBCL score by marital status were not significant at 5 and 9 years after adjusting for confounders. Parental marital status is associated with an increased CBCL internalizing behavior score at 3 years of age, but the association disappears at later time points.

## 1. Introduction

In the United States (U.S.), divorce rates have steadily increased from the 1950s through the 1970s, reaching a peak in 1981 [1]. Though divorce rates have since declined, marital disruption impacts an estimated 40-50% of married couples [2]. In addition to divorce, prior to marital dissolution, many couples experience destructive conflict that may be visible to and have an impact on children in the family [2].

Previous research has shown that marital disruption results in negative health outcomes for children and adolescents. A literature review reported that marital conflict or dissolution is associated with increased unintentional injuries and illnesses and a greater number of symptoms of poor physical health [3, 4]. The literature review also identified that children from divorced or high-conflict homes reported more aches and pains, circulatory system problems, and mental health issues [3]. In contrast, other studies have shown that parental divorce, as part of a group of events known as “loss and violence events,” had no relationship with adolescent or adult-onset depression [5]. The inconsistent findings and potential disparate outcomes for the impact of marital disruption on children's health indicate that further research in this area is needed.

To date, most research has focused on the impact of marital disruption on physical health or externalizing behaviors, such as attention-deficit/hyperactivity disorder (ADHD) or oppositional defiant disorder. Caregivers often find externalizing disorders more problematic because of the associated disruptive behaviors [6]; however, internalizing disorders such as depression and anxiety are also diagnosed in children. The prevalence of major depressive disorder (MDD) and anxiety disorders (i.e., obsessive compulsive disorder and posttraumatic stress syndrome) among children and adolescents ranges from 0.6 to 3.0% and from 2.2 to 9.5%, respectively [7].

Previous studies have shown that parental marital status is associated with internalizing disorders. One of the most comprehensive studies on the effects of parental marital status on behavior problems found that children were least depressed when living with both parents rather than a single parent [8]. However, this study also indicated that living with both biological parents when there is significant conflict resulted in worse psychological outcomes for children than living with single parents [8]. Additionally, a cohort study of children between the ages of 3 and 6 years found that children with separated/divorced/single parents were more likely to meet the preschool diagnostic criteria for MDD [9]. Lastly, research from a birth cohort in Christchurch, New Zealand, found that children with separated/divorced parents had an elevated risk of later conduct problems, mood disorder, and substance abuse [10].

Many studies point to family history of internalizing disorders as the strongest predictor of subsequent mental illness in children, but there is also evidence that stressful life events can act as a mediator of family history for severity of depressive symptoms in preschool children [9, 11]. Prior research has identified a possible mechanism for this relationship, demonstrating that each parent has a unique role to play in the development of a child's psychological wellbeing [12]. When one parent becomes a less visible participant in a child's life, the normal development of that child's mental health may be negatively impacted. Therefore, the current study aims to investigate the relationship between marital disruptions of parents and internalizing disorders of the child over a 5-year period.

## 2. Materials and Methods

Data from the Fragile Families and Child Wellbeing Study was utilized. The Fragile Families and Child Wellbeing Study is a cohort of approximately 5,000 children who were born in large U.S. cities between 1998 and 2000. Interviews were conducted with mothers and fathers at birth and when the child was one, three, five, and nine years old. All survey questions were aimed at collecting information on demographics and parenting behaviors, attitudes, and relationships. Additionally, an in-home assessment was conducted to obtain information on the child's cognitive and emotional development, health, and home environment [13]. Additional information on the Fragile Families and Child Wellbeing Study is available elsewhere [14]. The complete case analysis excluded families (identified through the family ID number) who did not respond to survey questions used to determine the marital status between the mother and father or did not respond to questions assessing depression and anxiety symptoms of the child at any time point, leaving 2,183 families.

The main exposure, marital status, was assessed at baseline using the following question, “Are you currently married to the father of your new baby?” Respondents could answer, “Yes, married to father,” “No, not married to father,” or “Father unknown.” When the child was three and five years old, the mother was asked, “What is your relationship with (FATHER) now? Are you… (married; romantically involved; separated/divorced; just friends; not in any kind of relationship; father not known; father died).” Marital status was then dichotomized (yes; no) at each time point.

The main outcome, child depression and anxiety indicators, was based upon validated survey questions adapted from the Child Behavior Checklist (CBCL). Research has demonstrated that the CBCL has high validity and reliability [15]. Moreover, research has shown that the anxiety and depression syndrome measure from CBCL is predictive of DSM-IV affective disorders [16]. The CBCL was completed by the primary caregiver when the child was three, five, and nine years of age. Caregivers provided a score for each question ranging from 0-2, with 0 representing a response of “not true,” 1 representing “somewhat/sometimes true,” and 2 representing “very/often true.” Because the CBCL questions varied in number and substance based on the age of the child, a standardized score for each time point was created by dividing the respondent's total score by the number of questions asked to the child. Higher scores of the CBCL indicate an increased likelihood for future health and behavioral problems.

A variety of factors identified in the literature were considered as potential confounders. Mother and father factors included age (continuous), race (non-Hispanic (NH) white; NH black; other), and education (less than high school; high school diploma or equivalent; some college or college graduate). Child factors included general health (excellent; very good; good; fair or poor). Due to a high amount of missing, income could not be assessed for the mother or father.

Descriptive statistics were calculated to obtain sample characteristics using percentages, frequencies, sample means, and standard errors. A repeated-measures multilevel regression model with a Toeplitz covariance structure was used to obtain Beta estimates and 95% confidence intervals. The Toeplitz covariance structure was selected by comparing the Akaike information criterion (AIC) between several common covariance structures. Further, the Kenward and Roger adjustment [17] was used to adjust for Type 1 error rate, which would otherwise be highly inflated [18]. Estimates were also compared between models to demonstrate balanced data, which signifies that estimates accurately show differences between means [19]. To adjust for multiple comparisons, Tukey's method was used for all analyses to maintain an overall significance level of *α* = 0.05. All analyses were conducted in SAS version 9.4 statistical software (PROC MIXED SAS 9.3 (Cary, N.C.)).

## 3. Results

Characteristics of the study population are summarized in Table 1. The majority of families were non-Hispanic black and reported excellent child health. Parents who were married at the child's birth were more likely to report non-Hispanic white race/ethnicity and had at least some college education. In contrast, unmarried parents were more likely to be non-Hispanic black and have less than a college education. Married mothers and fathers had mean ages of 30 and 32 years, respectively. Married and unmarried families differed in maternal and paternal age, race, education, and general health of the child.

The least squares mean estimates by marital status and child's age are shown in Table 2. Regardless of the parents' marital status, the CBCL internalizing score decreased at each time point. In unmarried families, the crude mean child CBCL score dropped from 0.37 at year 3 to 0.19 at year 9; in married families, the mean child CBCL score dropped from 0.27 to 0.16. At 3 years of age, internalizing scores were higher among children whose parents were not married, though the scores at 5 and 9 years were similar. After adjusting for race, education, maternal age, and general health of the child, the significant differences persisted and estimates strengthened.

Table 3 displays estimates of sequential time and overall differences by marital status. After adjusting for confounders, at 3 years of age, children in unmarried families had a 0.10 higher standardized CBCL score (95% CI: 0.09-0.12;* p*<.0001) compared to 3-year-old children in a married family; however, differences in CBCL score by marital status were not significant at 5 and 9 years. Significantly higher CBCL scores were found among children in unmarried families compared to married families over time. For example, children in unmarried families at 5 years of age had a 0.07 higher standardized CBCL score compared to children at 9 years of age in a married family. Lastly, the estimated correlation matrix (not shown) shows a high correlation with latter measurements which could indicate a reduction in the sum score of depression and anxiety at 5 years.

## 4. Discussion

Results from the current study suggest that parental marital status is associated with increased internalizing behaviors at 3 years of age; however, this association disappears as the child ages. Additionally, as the child ages, the CBCL score decreases for both the married and unmarried groups. Previous research has found behavioral problems in children who do not live with two biological parents [20]. Our results present a more nuanced association between parental marital status and child behavior, which suggests that the association exists at certain time points but not others.

The initial difference in CBCL scores by marital status could be explained in several ways. To start, the primary caregiver in most single-parent households is the mother [21]. Unmarried mothers frequently experience more hardship and less social support compared to married mothers [22]. Previous research has found that infant behaviors correlate with maternal feelings and behaviors [23]. In this instance, it is possible that the children of unmarried mothers mimic or are otherwise affected by the altered behavior of their mothers. Differences in child-care practices may also result in the observed difference by marital status, since unmarried mothers may not have anyone else in the home to assist in taking care of the child [22].

It is also possible that the higher CBCL scores in children with unmarried parents could be explained by differences in how married and unmarried parents perceive or report their child's behavior. Previous research has found that parental reports on their child's internalizing behavior can be affected by parental factors. Specifically, maternal psychological symptoms have been found to affect the mother's reporting of the child's internalizing behavior [24]. Previous research has found that single mothers are at a higher risk for depression and chronic stress [22] which may have led to increased reports of internalizing behaviors. However, this explanation does not explain why the CBCL scores were only different at age 3. There are a variety of factors which may influence the caregiver's report of internalizing disorders, and it is difficult to control for these potential confounders or speculate on their presence and effects in our analysis.

The relationship between marital status and child health may also be influenced by the home environment prior to the divorce or dissolution. In a paper based on the National Longitudinal Study on Adolescent Health, researchers found that the negative consequences for adolescents following a marital disruption were almost entirely explained by the level of conflict in the home prior to the disruption. In low-conflict marriages, children experienced more internalizing symptoms after disruption, while adolescents from households with high-conflict marriages experienced a decrease in behavioral problems [25].

This study had several strengths. First, the CBCL is a widely used, empirically tested scale for assessing behavioral problems in children and adolescents. Second, our sample was large which provided power to detect significant differences. The longitudinal design of the Fragile Families Survey enabled repeated-measures of marital status and internalizing disorders. In addition to the strengths of this analysis, there were also a few limitations. As previously mentioned, we could not control for differential reporting of child behavior in married and unmarried families. Longitudinal weights were not available; therefore, results are not representative of the original sample and our results do not generalize to the entire population [26]. Score comparisons between time points may be obscured by the CBCL methodology which uses different questions (in wording and number) for 2-5 year olds and 6-18 year olds. However, we attempted to overcome this limitation by using a standardized score instead of the total score. Finally, we were unable to account for possible cohabitation in families, contact between the infant and parents, and the presence of any other parent-figures in the child's life.

## 5. Conclusion

Parental marital status is associated with an increased CBCL internalizing behavior score at 3 years of age, but the association disappears at later time points. It remains to be seen if this initial increase is predictive of behavioral problems in late adolescence or beyond. Children in families with unmarried parents are known to have increased risk for behavioral problems, but this association may only manifest itself during certain developmental periods in childhood. Future research is needed to understand child and adolescent outcomes associated with an increased CBCL score at a young age.

## Figures and Tables

**Table 1:** Distribution of family characteristics by marital status at baseline.

	Total	Married	Not Married	*p*-value
	%	%	%	
*Maternal age (mean; se)*	25.1 (0.1)	29.5 (0.2)	23.7 (0.1)	
*Maternal race*				<.0001
White, Non-Hispanic	30.2	55.1	22.0	
Black, Non-Hispanic	53.5	26.4	62.4	
Hispanic/Other	16.3	18.6	15.6	
*Maternal education*				<.0001
Less than high school	36.8	15.1	43.9	
High school	26.4	16.5	29.6	
At least some college	36.9	68.4	26.6	
*Paternal age (mean; se)*	27.7 (0.2)	31.9 (0.3)	26.0 (0.2)	
*Paternal race*				<.0001
White, Non-Hispanic	19.8	48.6	10.4	
Black, Non-Hispanic	54.2	27.0	63.1	
Hispanic/ Other	26.0	24.4	26.5	
*Paternal education*				<.0001
Less than high school	31.5	13.8	37.5	
High school	36.4	23.2	40.9	
At least some college	32.1	63.1	21.7	
*Child gender*				0.7735
Male	51.9	52.5	51.8	
Female	48.1	47.5	48.2	
*General health (Child)*				0.0003
Excellent	57.8	65.0	55.5	
Very good	28.0	25.5	28.8	
Good	11.6	8.1	12.8	
Fair/poor	2.5	1.5	2.9	

*Note*. SE= standard error; y.o=years old.

**Table 2:** Least squares mean estimates and standard errors by marital status and child's age.

Marital Status	Child's Age	Estimate	*p-*value
	(years)	(SE)	
Crude			

Not Married	3	0.37 (0.01)	<.0001
	5	0.26 (0.01)	<.0001
	9	0.19 (0.01)	<.0001

Married	3	0.27 (0.01)	<.0001
	5	0.21 (0.01)	<.0001
	9	0.16 (0.01)	<.0001

Adjusted^a^			

Not Married	3	0.39 (0.01)	<.0001
	5	0.29 (0.01)	<.0001
	9	0.22 (0.01)	<.0001

Married	3	0.32 (0.01)	<.0001
	5	0.27 (0.01)	<.0001
	9	0.22 (0.01)	<.0001

*Note*. SE= standard error.

^a^Adjusted for maternal age, race, and education, paternal race and education, and general health of the child.

**Table 3:** Estimates of sequential time point and overall differences by marital status.

Marital Status Comparison	Time Comparison	Estimate	SE	95% CI	t	*p*_*adj*_
Crude						

Married vs. Married	3 vs. 5	0.05	0.01	0.03-0.08	5.57	<.0001
	3 vs. 9	0.11	0.01	0.07-0.14	9.46	<.0001
	5 vs. 9	0.06	0.01	0.03-0.08	5.52	<.0001

Married vs. N. Married	3 vs. 5	0.004	0.01	-0.02-0.03	0.41	0.9985
	3 vs. 9	0.07	0.01	0.45-0.10	7.30	<.0001
	5 vs. 9	0.02	0.01	-0.01-0.05	2.31	0.1918

N. Married vs. Married	3 vs. 3	0.10	0.01	0.07-0.13	10.13	<.0001
	5 vs. 5	0.05	0.01	0.02-0.08	4.76	<.0001
	9 vs. 9	0.03	0.01	0.00-0.06	2.87	0.0480

N. Married vs. Married	3 vs. 5	0.15	0.01	0.12-0.18	15.31	<.0001
	3 vs. 9	0.21	0.01	0.18-0.24	19.44	<.0001
	5 vs. 9	0.10	0.01	0.07-0.13	9.60	<.0001

N. Married vs. N. Married	3 vs. 5	0.10	0.01	0.09-0.12	17.48	<.0001
	3 vs. 9	0.18	0.01	0.15-0.20	23.75	<.0001
	5 vs. 9	0.07	0.01	0.05-0.09	10.86	<.0001

Adjusted^a^						

Married vs. Married	3 vs. 5	0.05	0.01	0.03-0.08	5.62	<.0001
	3 vs. 9	0.10	0.01	0.07-0.14	9.27	<.0001
	5 vs. 9	0.05	0.01	0.02-0.08	5.25	<.0001

Married vs. N. Married	3 vs. 5	0.03	0.01	-0.001-0.06	2.80	0.0573
	3 vs. 9	0.10	0.01	0.07-0.13	8.82	<.0001
	5 vs. 9	0.04	0.01	0.01-0.08	4.10	0.0006

N. Married vs. Married	3 vs. 3	0.10	0.01	0.09-0.12	16.89	<.0001
	5 vs. 5	0.02	0.01	-0.01-0.05	2.08	0.2986
	9 vs. 9	0.01	0.01	-0.03-0.04	0.68	0.9838

N. Married vs. Married	3 vs. 5	0.13	0.01	0.09-0.16	11.79	<.0001
	3 vs. 9	0.18	0.01	0.15-0.21	15.72	<.0001
	5 vs. 9	0.07	0.01	0.05-0.09	10.03	<.0001

N. Married vs. N. Married	3 vs. 5	0.10	0.01	0.09-0.12	16.89	<.0001
	3 vs. 9	0.17	0.01	0.15-0.19	22.60	<.0001
	5 vs. 9	0.07	0.01	0.47-0.09	10.03	<.0001

*Note*. SE= standard error.

^a^Adjusted for maternal age, race, and education, paternal race and education, and general health of the child.

## Data Availability

The Fragile Families and Child Wellbeing Study data used to support the findings of this study are publicly available from Princeton University's Office of Population Research data archive.
